# Out of distribution detection with attention head masking for multimodal document classification

**DOI:** 10.1038/s41598-025-32328-9

**Published:** 2026-01-03

**Authors:** Christos Constantinou, Georgios Ioannides, Aman Chadha, Aaron Elkins, Edwin Simpson

**Affiliations:** 1 University of Bristol, Bristol, England; 2 James Silberrad Brown Center for Artificial Intelligence, San Diego State University, San Diego, USA; 3 Carnegie Mellon University, Pittsburgh, USA; 4 Stanford University, Stanford, USA; 5 Amazon GenAI (Work does not relate to position at Amazon), Amazon, London, United Kingdom; 6 Amazon GenAI (Work does not relate to position at Amazon), Amazon, San Diego, USA; 7 Amazon GenAI (Work does not relate to position at Amazon), Amazon, San Francisco, USA

**Keywords:** Engineering, Mathematics and computing, Computational science

## Abstract

Detecting out-of-distribution (OOD) data is critical for ensuring the reliability and safety of deployed machine learning systems by mitigating model overconfidence and misclassification. While existing OOD detection methods primarily focus on uni-modal inputs, such as images or text, their effectiveness in multi-modal settings, particularly documents, remains underexplored. Moreover, most approaches prioritize decision mechanisms over optimizing the underlying dense embedding representations for optimal separation. In this work, we introduce Attention Head Masking (AHM), a novel technique applied to Transformer-based models for both uni-modal and multi-modal OOD detection. Our empirical results demonstrate that AHM enhances embedding quality, significantly improving the separation between in-distribution and OOD data. Notably, our method reduces the false positive rate (FPR) by up to 10%, outperforming state-of-the-art approaches. Furthermore, AHM generalizes effectively to multi-modal document data, where textual and visual information are jointly modeled within a Transformer architecture. To encourage further research in this area, we introduce FinanceDocs, a high-quality, publicly available document AI dataset tailored for OOD detection. Our code and dataset is available at https://github.com/constantinouchristos/OOD-AHM.

## Introduction

Out-of-distribution (OOD) detection presents a significant challenge in the field of multi-modal document classification. When a classifier is deployed, it may encounter types of documents that were not included in the training dataset. This can lead to mishandling of such documents, causing additional complications in a production environment.

**Fig. 1:**
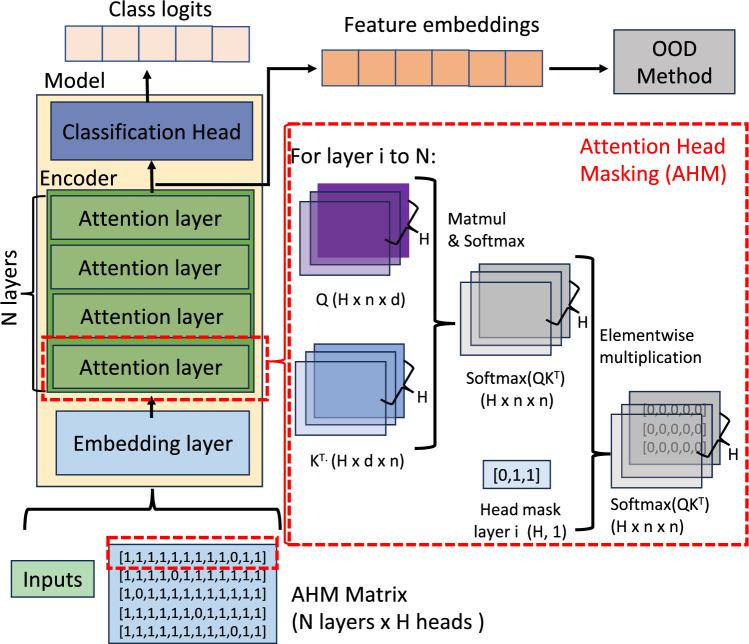
Visual demonstration of AHM on a transformer-based model: For each attention layer, we utilize the corresponding attention head mask from the AHM matrix. Following query-key multiplication and the subsequent softmax operation, the resulting attention scores undergo element-wise multiplication with the relevant attention head mask. This process effectively reduces the attention scores of certain heads to zero, thereby inhibiting the propagation of their respective information through the value matrix.

**Fig. 2:**
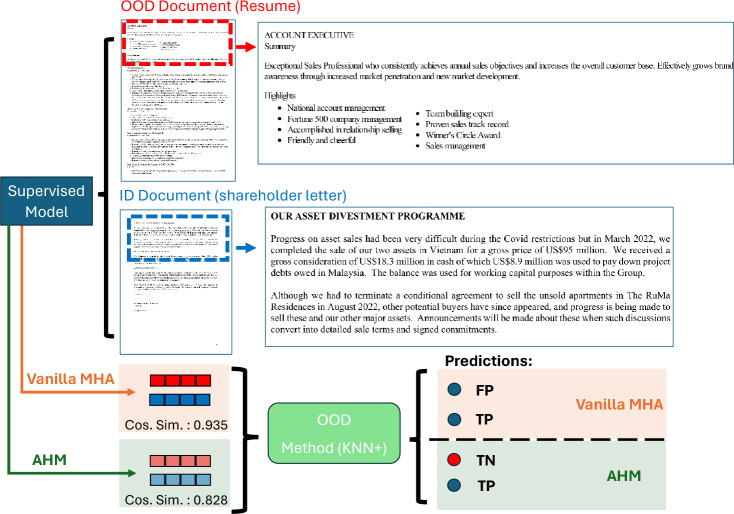
Illustration of the effectiveness of AHM in reducing false positive rates on real-world samples. The blue-highlighted example represents an in-distribution (ID) class, “shareholder letter”, while the red-highlighted example corresponds to an out-of-distribution (OOD) class, “resume”. This sample pair demonstrates a case where low similarity between the embedding representations of ID and OOD data is expected. The orange path illustrates the embeddings and corresponding predictions obtained using standard Multi-Head Attention (Vanilla MHA), whereas the green path represents the embeddings and predictions generated when Attention Head Masking (AHM) is applied.

Effective OOD detection facilitates the identification of unfamiliar documents, enabling the system to manage them appropriately to maintain its reliability and accuracy in real-world applications. In OOD detection, the primary objective is to determine if a new document belongs to a known in-distribution (ID) class or an OOD class. A significant challenge lies in the lack of supervisory signals from the unknown OOD data, which can encompass any content outside the ID classes. The complexity of this problem increases with the semantic similarity between the OOD and ID data^1^.

The confusion between OOD and ID data, particularly when they exhibit high semantic similarity, may stem from the model’s reliance on a limited set of features that are sufficient to distinguish between the known ID classes, but may not differentiate ID classes from novel OOD classes. This problem is illustrated in Fig. 2, demonstrating the dense embedding representations of an in-distribution (ID) document—a shareholder letter (blue) and an out-of-distribution (OOD) document—a résumé (red). Using Vanilla Multi-Head-Attention (orange path), the embeddings of these documents exhibit a high cosine similarity of 0.935, likely due to both documents discussing sales, which activates similar feature extractors in the model architecture. Since the model relies on a limited set of dominant features, the extracted embeddings become highly similar, leading to a false positive OOD prediction (blue).

Techniques such as MC Dropout^2^, which approximate Bayesian neural networks to estimate model uncertainty, can be employed in OOD detection by randomly deactivating neurons during inference. This mechanism allows the model to assess multiple variations in feature representations rather than depending on a single, fixed representation. However, MC Dropout primarily affects a small portion of neurons within the feature extraction layers, causing relatively small changes to the feature representations. This makes it less effective at suppressing features that are similar between ID and OOD data, and hence in ensuring that the model considers diverse features.

To address this limitation, our proposed Attention Head Masking (AHM) method selectively masks entire attention heads rather than just individual neurons, as illustrated in Fig. 1. AHM constructs an ensemble of embeddings by averaging representations obtained from multiple masked configurations selected to maximize similarity between samples from the same class. This selective masking encourages the model to diversify its learned feature representations, reducing overreliance on a small set of dominant features that may exhibit high semantic overlap with OOD data. Empirical results demonstrate that AHM improves OOD detection robustness by enhancing feature diversity, ultimately mitigating the risk of confusing semantically similar ID and OOD instances. The effectiveness of AHM is illustrated in Fig. 2 (green path) where AHM masks different attention heads, forcing the model to utilize a more diverse set of feature extractors. Consequently, when embeddings are computed with AHM during inference, their similarity decreases, resulting in a lower cosine similarity score of 0.828. This enables the downstream OOD detection method to correctly classify the OOD instance as OOD or a true negative (red), reducing the false positive rate.

While numerous OOD detection techniques have been proposed, most have been evaluated solely on uni-modal systems, such as text^3^ or images^4^, with limited exploration in the document domain^5^. A key challenge for multi-modal OOD detection is the scarcity of high-quality, publicly available document datasets, as most existing work relies on IIT-CDIP^6^. To bridge this gap, we introduce FinanceDocs, a new multi-modal document AI dataset.

Our key contributions are:

- **FinanceDocs dataset**: A high-quality, multi-modal document dataset tailored for OOD detection, offering digital PDFs instead of degraded scans.
- **AHM for OOD detection**: A novel post-fine-tuning attention masking technique that improves feature robustness and enhances ID-OOD separation in Transformer models.
- **State-of-the-art performance**: AHM outperforms existing OOD methods, reducing the false positive rate (FPR) by up to 10% across uni-modal text and multi-modal document settings.

## Related work

Out-of-distribution (OOD) research spans multiple subfields, including efforts aimed at enhancing model performance to unseen data distributions—commonly referred to as OOD generalization. For instance^7^, introduced a Mutual-enhanced Incongruity Learning Network (MILNet) for multi-modal sarcasm detection, which promotes generalization to incongruent or noisy inputs by enforcing cross-modal consistency through mutual learning between global and local incongruity modules, selective knowledge transfer, and semantic relation modeling. Similarly^8^, proposed a generative multimodal sarcasm detection model that enhances generalization to unseen distributions by incorporating a visual instruction template and a demonstration retrieval mechanism, enabling the model to learn broader semantic and visual patterns rather than overfitting to domain-specific cues. Further work on OOD generalisation in a multi modal setting includes CF-VQA^9^, CLUE^10^ and GEAR^11^. CLUE and GEAR demonstrated that models over-rely on spurious corelations in textual modality, leading to degraded performance on OOD test sets. CLUE utilizes counterfactual inference to disentangle direct and indirect textual effects, while GEAR employs inverse probability weighting to reduce reliance on biased features. Similarly, CF-VQA addresses language bias in Visual Question Answering through causal effect decomposition.

These findings motivate our work on OOD detection: if multimodal models are prone to failure under distribution shift, it becomes critical to detect when the inputs fall outside the training distribution. However, our problem formulation differs fundamentally-rather than improving classification performance under distribution shift, our work addresses the complementary line of research that focuses on OOD detection, which seeks to identify inputs that deviate from the training distribution at deployment time. Consequently, our evaluation uses detection metrics (AUROC, FPR) rather than classification accuracy. Despite progress in OOD generalization, OOD detection remains essential because real-world systems frequently encounter inputs belonging to *previously unseen or entirely unknown classes*, for which generalization-based methods offer no guarantee of correct behavior. This necessity further motivates the development of detection-focused approaches.

Although our objective differs from CF-VQA^9^, CLUE^10^, and GEAR^11^, our proposed method shares an important conceptual link: reducing the influence of dominant, spurious features. In our case, Attention Head Masking (AHM) suppresses misleading attention heads that bias the representations, thereby enhancing sensitivity to distributional deviations and improving reliability in deployment. The novelty of our work lies in introducing AHM, a simple attention-masking mechanism that can be integrated into a broad range of attention-based architectures —distinct from OOD generalization methods—and demonstrating consistent improvements in OOD detection metrics across both unimodal and multimodal settings.

**Fig. 3:**
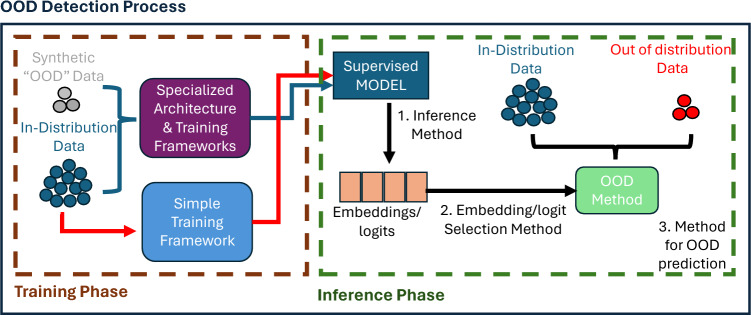
Schematic of OOD optimization methods in OOD Process. Training and Inference Phases. There are three stages in the Inference Phases of OOD process. Stage one of the process explores different inference methods for generating logits or embeddings from the fine-tuned model. Stage two focuses on how to effectively aggregate the generated logits or embeddings. Stage three is the actual OOD method that creates the decision boundary between ID and OOD data using the outputs of stage two.

Numerous approaches have been proposed to address the task of out-of-distribution (OOD) detection. To provide a clearer understanding of how different research directions contribute to optimizing OOD detection performance, we illustrate the overall OOD process in Fig. 3. We categorize existing research into two primary areas: (A) approaches that improve OOD detection by introducing modifications to training frameworks, model architectures, or synthetic OOD data generation strategies “Training Phase” methods^12–16^, and (B) approaches that enhance OOD detection at inference time while maintaining standard training pipelines “Inference Phase” methods^3,17^. Our study focuses on the latter category, specifically the inference-phase methods, which we further decompose into three stages as illustrated in Fig. 3.

After a model has been fine-tuned on supervised in distribution (ID) data it has effectively gained knowledge of the underlying features of the ID data. This knowledge can be subsequently utilized in the process shown in Fig. 3 to develop an OOD detection system. Stage one of the process explores different inference methods for generating logits or embeddings from the fine-tuned model. Stage two focuses on how to effectively aggregate the generated logits or embeddings from different layers of the model and thus create the final input features for different OOD decision strategies in stage three. Finally, stage three focuses on the methods that create decision boundaries between ID and OOD data.

Early research for the OOD task heavily focused on stage three which is the actual OOD decision method in an OOD system. A number of OOD decision methods have been developed to differentiate OOD data from ID data, broadly classified into three categories: (i) confidence-based methods, which focus on softmax confidence scores^18–21^, (ii) features/logits-based methods, which combine information from logits with intermediate features^22–26^, and (iii) distance/density-based methods, which concentrate on dense embeddings from the final layers^27–29^.

Recent research also investigates domain-invariant representations, such as HYPO^30^, and introduces new OOD metrics like NECO^31^, which leverage neural collapse properties^32^. Confidence-based methods can be unreliable as they often yield overconfident scores for OOD data^18^. Features/logits-based methods which attempt to compute OOD scores by combining logits, which depend on the ID classes, with features, which are class-agnostic. Our approach focuses on identifying more robust class-agnostic scores from the feature space, and as such, we conduct our experiments using distance/density-based methods.

As shown in Fig. 3, a critical component of the OOD process is the extraction of logits or embeddings in stage one. Learning embedding representations that generalize well and effectively differentiate between ID and OOD data is a well-recognized challenge^33^, with a significant impact on OOD performance^1^. Various studies have addressed this by optimizing intra-class compactness and inter-class separation^34^. These studies predominantly focus on optimizing the ’Training Phase’ of the OOD process depicted by the blue arrow path in Fig. 3. One prominent approach is Prototypical Learning (PL), which builds on contrastive learning principles. Methods such as^35^ and^36^ employ prototypes derived from offline clustering and pull the representations of data points around their nearest prototypes, to enhance the separability of classes and generalizability of representations across clusters. More recently,^30^ integrated PL into their OOD detection framework, HYPO, demonstrating effective ID-OOD separation. This was further extended by^37^, who introduced multiple prototypes per cluster and used a maximum likelihood estimation (MLE) loss to refine feature alignment. In parallel, other recent methods have explored alternative training objectives and model formulations to enhance OOD separability.^38^ leverage diffusion models^39^ in the latent representation space to compute semantically meaningful likelihoods, thereby improving the alignment between learned representations and OOD decision boundaries.^15^ introduce an evidential learning framework where the output layer predicts parameters of a Beta distribution instead of traditional logits, allowing the model to express calibrated uncertainty on a per-class basis using an evidence-based loss . Another approach by^13^ introduces an auxiliary “garbage” class, trained via inverted representations, to explicitly capture OOD signals and isolate them from ID distributions. Complementary to these strategies,^14^ propose a hybrid training objective that jointly optimizes the standard cross-entropy loss with a TRADES-style robust loss^40^, encouraging smoother decision boundaries that generalize more effectively to unseen OOD samples.While these methods enhance embedding quality and OOD separability, they require significant training modifications. For example,^38^ rely on diffusion-based architectures, limiting general applicability, and show weaker performance than classical methods like Mahalanobis under label supervision.^15^ introduce evidential losses with Beta distributions, adding model complexity and calibration challenges. Other approaches^13,14^ depend on auxiliary classes or robust losses, which require careful tuning and may hinder scalability as they diverge from standard cross-entropy loss.

Other techniques focus on generating synthetic OOD samples to improve decision boundaries. Methods such as VOS^41^ and NPOS^16^ explicitly generate OOD-like representations to refine feature spaces. Similarly,^42^ leverage external open-source data as an OOD signal. However, our study assumes no access to OOD data and does not rely on synthetic OOD generation. Instead, we focus on enhancing the representation of embeddings directly during inference.

Unlike the aforementioned approaches that require synthetic data^13,41^, or specialized architectures^15^, our AHM method improves ID-OOD separation purely at inference time, operating within stage one of the OOD process. Instead of introducing new auxiliary objectives^13^ or clustering-based training objectives (^30,36,37^), we propose attention head masking as a feature regularization technique. This ensures robust OOD detection without incurring additional training costs or potential performance degradation, as the method can be applied to existing pretrained models at inference time.

Other studies explored embedding aggregation techniques at stage two of the OOD process. Early methods relied solely on the CLS token embedding, commonly used in models such as BERT^43^, RoBERTa^44^, and LayoutLMv3^45^. Later work, such as Avg-Avg^3^ and Gnome^17^, sought to enhance sequence-level representations for OOD detection. Avg-Avg averages embeddings across both sequence length and layers, while Gnome combines embeddings from pre-trained and fine-tuned models to improve generalization.

While these approaches emphasize embedding manipulation during inference—similar to our work—they primarily operate in stage two, focusing on how embeddings are aggregated rather than how they are generated. In contrast, AHM directly influences the initial embedding generation process (stage one), ensuring stronger feature separation before aggregation occurs.

Another technique that operates at stage one of the OOD process is MC Dropout^2^, which applies Dropout^46^ at inference time to estimate model uncertainty. By randomly masking a portion of neurons, MC Dropout effectively generates an ensemble of predictions, enhancing feature diversity. While MC Dropout shares similarities with our proposed AHM method—both involve feature suppression during inference—it differs in key aspects. MC Dropout operates at the neuron level, stochastically shutting down a fixed percentage of neurons within a layer. In contrast, AHM operates at the feature extractor level, selectively masking entire attention heads in the multi-head attention mechanism. This provides greater control and flexibility, allowing for both random and targeted masking strategies to selectively shut down features and thus optimize performance. Furthermore, because MC Dropout only removes a small subset of neurons, it remains limited in its ability to suppress dominant but misleading features that cause confusion between ID and OOD data. In contrast, AHM more effectively diversifies feature representations, leading to better ID-OOD separation and improved robustness in OOD detection systems.

## Method

The proposed AHM method focuses on stage one of the OOD process (Fig. 3) and depends on the feature extraction mechanisms inherent in transformer models, specifically the self-attention mechanism^47^. Based on the premise that OOD data exhibit less semantic similarity to ID data, our goal is to generate embedding features that enhance the separation between ID and OOD data. A demonstration of one AHM step is summarised in Fig. 1.

The embeddings are then used in stage three of the OOD process along with a distance or density-based OOD detection method, such as the Mahalanobis^28^ or kNN+^29^. The Mahalanobis models in-distribution (ID) feature distributions as class-conditional Gaussians by estimating their mean and covariance. For a test sample, the Mahalanobis distance to the closest Gaussian is computed, with a threshold applied to classify it as ID or OOD. KNN+ extends k-nearest neighbors (KNN) by incorporating local density information. It computes the distances between a test sample and its k-nearest ID neighbors, refining OOD scores to account for distribution variations. A threshold is then applied to classify the sample as ID or OOD.

### Theoretical framework overview

The central assumption underlying the proposed solution is that ID data should exhibit greater similarity in their feature representations to each other than to OOD data. Consequently, given a pair of data points from two similar ID classes (Pair A) and a pair consisting of one ID and one OOD data point (Pair B), a masking procedure applied to feature representations should result in a more pronounced divergence for Pair B than for Pair A. This follows from the assumption that OOD samples, lacking alignment with learned ID features, should undergo greater representational shifts when certain feature extraction pathways are suppressed.

A key challenge in OOD detection is that models may over-rely on specific *class-activating* features that drive ID classification, but do not discriminate OOD from ID data, leading to misclassification of semantically similar OOD data. Our hypothesis is that by selectively suppressing certain features, the model will be encouraged to rely on more diverse feature sets, thereby enhancing separation between ID and OOD data. We consider two critical ways to influence the final feature embeddings used in distance-based OOD detection methods: through (a) the input tensors provided to the model or (b) the feature extraction mechanism, specifically the attention mechanism.

A straightforward approach to suppressing features is input masking, where parts of the input (textual or visual) are obscured. In textual data, this involves replacing tokens randomly with the [MASK] token, while in visual data, selected image patches are zeroed out. Although input masking can effectively remove information, it presents challenges in ensuring effective suppression of dominant features during inference. In visual data, masking can be performed by dividing an image into uniform patches, which can be masked to suppress features. However, this approach does not guarantee the masking of class-activating regions, as some masked areas may contain little discriminative information. Optimizing the mask is made difficult because the importance of each region of the image can differ between individual samples (e.g., if one image contains more whitespace or uses a different zoom level). For textual data, the position of class-activating information is also highly variable, but sequence length variability poses an additional challenge when choosing an effective mask: Applying a consistent mask across multiple documents may inadvertently suppress non-informative tokens, such as padding, rather than the dominant class-activating features. These limitations reduce the effectiveness of input masking as a method for suppressing dominant features in OOD detection.

To overcome these limitations, we focus instead on the feature extraction process itself, particularly the attention mechanism within Transformer-based models. Instead of modifying the input, we achieve controlled suppression by selectively masking attention heads within different encoder layers. These attention heads capture distinct representations of the input, learning various patterns and relationships. By deactivating specific heads, we effectively suppress certain feature extraction pathways in a structured and consistent manner—regardless of variations in the input data—, ensuring a more stable and effective method for feature suppression which helps enhance OOD detection mechanisms.

### Theoretical analysis of attention head masking (AHM)

We provide a theoretical perspective to explain why Attention Head Masking (AHM) improves ID–OOD separability, complementing our empirical findings. For simplification we consider AHM within a single layer however the same concept is applied across all layers where our proposed AHM is applied.

Feature Subspace View. Let the embedding produced by a Transformer be expressed as a sum over attention heads:

$$\begin{aligned} \phi (x) = \sum _{h=1}^{H} W_h f_h(x), \end{aligned}$$

where $f_h(x)$ is the feature extracted by head *h*, and $W_h$ is a projection matrix. Each $f_h(x)$ lies in a subspace $S_h \subseteq \mathbb {R}^d$. In practice, a small subset of heads dominate because their features align strongly with class-discriminative signals in the in-distribution (ID) training data. However, these “dominant features” may not distinguish ID from OOD, especially when OOD inputs share superficial similarities with ID classes.

AHM constructs masked embeddings

$$\begin{aligned} \phi _M(x) = \sum _{h \in M} W_h f_h(x), \end{aligned}$$

for a subset of heads $M \subseteq \{1, \dots , H\}$. The final AHM embedding is obtained by averaging across masks:

$$\begin{aligned} \bar{\phi }(x) = \frac{1}{|\mathcal {M}|}\sum _{M \in \mathcal {M}} \phi _M(x), \end{aligned}$$

where $\mathcal {M}$ denotes the set of selected masks. This process has two effects: (1) it reduces the influence of any single dominant subspace, and (2) it forces embeddings to retain discriminative power across multiple complementary subspaces.

Variance-Based Separation. Consider the embedding variance across masks,

$$\begin{aligned} \sigma ^2(x) = \mathbb {E}_{M}\Big [\Vert \phi _M(x) - \bar{\phi }(x)\Vert ^2\Big ]. \end{aligned}$$

For ID samples, features are well-aligned with multiple heads, so $\sigma ^2(x)$ remains low. For OOD samples, alignment is weaker and less consistent across head subsets, yielding larger $\sigma ^2(x)$. Consequently, the distributions of ID and OOD embeddings become more separable. This phenomenon is consistent with ensemble variance-reduction theory, which shows that averaging over diverse components reduces variance for well-aligned signals while exposing instability in misaligned ones^48^. In our setting, AHM stabilizes ID representations through averaging across attention subspaces, while amplifying uncertainty in OOD representations, thereby improving separability.

Connection to Distance-Based OOD Methods. In distance-based detection (e.g., Mahalanobis, kNN), separability depends on the margin between ID and OOD embeddings. Let $\mu _{ID}$ and $\mu _{OOD}$ denote mean embeddings. With AHM,

$$\begin{aligned} \Vert \mu _{ID} - \mu _{OOD}\Vert \quad \uparrow , \end{aligned}$$

since $\mu _{ID}$ is preserved (low variance) while $\mu _{OOD}$ drifts due to inconsistent head activations. Thus, AHM enlarges the effective ID–OOD margin without requiring retraining.

Multi-Modal Implications. In multi-modal Transformers, attention heads specialize in different roles: some capture intra-modal dependencies (text–text or image–image), while others capture cross-modal alignments (text–image). Without masking, embeddings often over-rely on text-dominant heads, as text carries dense semantic information. This bias leads to OOD errors when OOD text resembles ID text, even if visual or layout cues differ (e.g., résumés vs. shareholder letters Fig. 2) .

By selectively masking subsets of heads, AHM enforces greater utilization of cross-modal heads. The resulting embeddings integrate a richer combination of text, layout, and image cues, thereby reducing modality imbalance. Formally, if $H_T, H_V, H_{TV}$ denote text, visual, and cross-modal head sets, then AHM ensures that

$$\begin{aligned} \bar{\phi }(x) \approx \alpha _T \phi _{H_T}(x) + \alpha _V \phi _{H_V}(x) + \alpha _{TV} \phi _{H_{TV}}(x), \end{aligned}$$

with balanced contributions $\alpha _T, \alpha _V, \alpha _{TV}$, rather than being dominated by $\alpha _T$. This balanced representation explains why AHM yields stronger separability in multimodal settings.

### Choice of attention head masks

Our proposed method, AHM, applies feature suppression to create multiple diverse subsets of features, while retaining essential information.

With AHM, the aim is to selectively identify attention head masks that withhold some features but do not exclude all important features, so that we preserve high semantic similarity between ID data. To avoid random masking, which risks turning off such features, we follow the steps detailed in Algorithm 1.

We begin with a fine-tuned model and proceed by randomly initializing various attention head masks based on a masking hyperparameter $p$. This hyperparameter represents the percentage of attention heads $H$ set to zero within each attention layer $N$ of the model. For each random mask, we extract dense hidden representations from both the training and evaluation datasets. The objective is to identify which of these randomly generated attention head masks minimizes the divergence between the representations of the evaluation and training data in the feature space. This is accomplished by calculating the average similarity score among the top $K$ nearest neighbors for each evaluation data point. The attention head masks are then ranked based on these aggregated similarity scores. Finally, we select the top $F$ masks with the highest similarity scores between the evaluation and training data and use them to generate new feature representations. These features are then ensembled (i.e., averaged) and subsequently utilized in a distance-based OOD detection method, such as the Mahalanobis distance.

**Algorithm 1:**
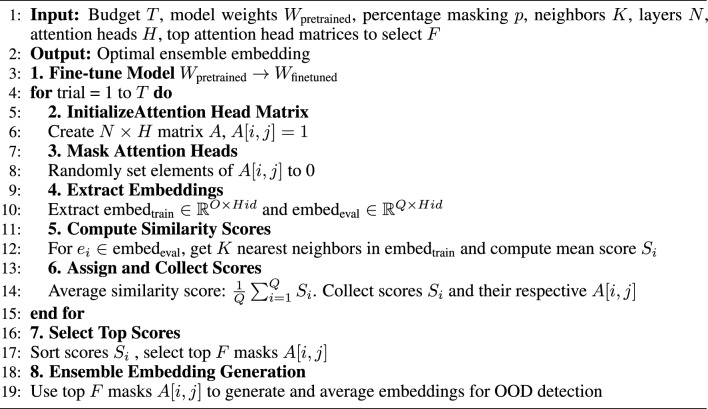
Identification of optimal of attention head masks for OOD detection.

### Computational efficiency and scalability

**Inference complexity:** As with any ensemble-based method, the computational requirements at inference time are proportional to the number of representations being aggregated. Specifically, our method has a complexity of $\mathcal {O}(K \times T)$, where *K* is the number of AHM configurations selected via Algorithm 1 and *T* is the time for a single forward pass through the transformer model. For each input, *K* feature embeddings are generated using the corresponding *K* AHM masks and are subsequently averaged to produce the final representation used in downstream OOD prediction. This linear scaling with respect to ensemble size is consistent with other ensemble-based OOD detection methods.

**Offline optimization complexity:** The primary computational cost lies in the offline search phase of Algorithm 1, which has a complexity of $\mathcal {O}(N \times M \times E)$, where *N* is the number of randomly sampled candidate AHM configurations, *M* is the size of the in-distribution validation dataset, and *E* is the time to evaluate similarity preservation for one configuration. This involves randomly sampling candidate AHM configurations and evaluating them on in-distribution data to identify those that best preserve semantic similarity. While this step introduces additional pre-processing overhead, it is a one-time operation performed before deployment and does not affect runtime performance. Future work may explore more efficient strategies such as gradient-based optimization or learned selection mechanisms to reduce this cost from $\mathcal {O}(N)$ to potentially $\mathcal {O}(\log N)$.

## Experiments

We conduct multi-modal and uni-modal experiments to explore the effectiveness of our proposed AHM against other OOD techniques that are applied at the three different stages of the OOD process.

### Datasets

For our multi-modal experiments we utilized two datasets: Tobacco3482 and FinanceDocs. The Tobacco3482 dataset^49^ comprises 10 classes: Memo (619), Email (593), Letter (565), Form (372), Report (261), Scientific (255), Note (189), News (169), Advertisement (162), and Resume (120). As a subset of IIT-CDIP^6^, it was further processed to remove blank and rotated pages, preserving the rich textual and image modalities essential for a multi-modal system. Despite these efforts, some instances exhibit poor OCR quality due to the low-quality scans.

We present FinanceDocs (cf. Appendix A.3 for per-category details and A.5 Figures S2–S11 for dataset samples), a newly created dataset comprising 10 classes derived from open-source financial documents, including SEC Form 13 (663), Financial Information (360), Resumes (287), Scientific AI Papers (267), Shareholder Letters (256), List of Directors (188), Company 10-K Forms (181), Articles of Association (176), SEC Letters (141), and SEC Forms (121). Unlike Tobacco3482, FinanceDocs consists of high-quality digital PDFs^50–54^. Furthermore, the selected document classes were chosen to ensure semantic similarity across multiple modalities—text, layout, and image—within the in-distribution (ID) set, thereby creating a more challenging scenario for OOD detection. As illustrated in A.5, resumes exhibit visual characteristics that closely resemble those of articles of association. Similarly, as shown in Fig. 2, resumes may share textual similarities with shareholder letters.

The FinanceDocs dataset was labeled through the following process: a PDF parsing package (PyPDF2) was used to extract content from the original PDF documents. Each page was then visualized individually by a human annotator, who determined the relevance of the page to the collected classes and assigned the appropriate class label (cf. Appendix A.2 for annotator training and validation).

To further explore the effectiveness of our proposed method we also conduct uni-modal experiments with three widely explored datasets namely SST-2^55^ and IMDB^56^ for sentiment analysis as well as 20-Newsgroups^57^ for topic classification.

**Table 1:** Multi-modal Performance metrics process stage 3 (percentage values; arithmetic mean and standard deviation) for different methods across two datasets with intra-dataset and cross-dataset experiments using AUROC (higher is better) and FPR (lower is better)– (cf. Appendix A.1 for hyperparameter tuning details).

Method	Tobacco3482 (ADVE OOD)		Tobacco3482 (Cross-dataset OOD)		FinanceDocs (Resume OOD)		FinanceDocs (Cross-dataset OOD)	
	AUROC (%)	FPR (%)	AUROC (%)	FPR (%)	AUROC (%)	FPR (%)	AUROC (%)	FPR (%)
energy	95.1 ± 1.2	26.7 ± 5.7	94.4 ± 1.4	15.7 ± 4.2	84.8 ± 9.3	41.3 ± 21.8	84.6 ± 1.6	56.7 ± 3.9
gradNorm	94.0 ± 2.5	33.0 ± 11.6	82.4 ± 4.0	41.0 ± 9.4	74.2 ± 15.3	66.4 ± 25.1	72.4 ± 12.8	81.7 ± 14.5
kl	91.4 ± 1.6	44.8 ± 9.9	97.0 ± 1.4	7.1 ± 3.5	90.2 ± 4.0	29.5 ± 10.6	84.0 ± 2.5	63.0 ± 4.7
knn	95.8 ± 1.1	26.9 ± 7.4	99.1 ± 0.4	3.0 ± 1.8	96.5 ± 2.3	17.2 ± 12.7	89.1 ± 1.7	58.9 ± 6.7
maxLogit	94.6 ± 1.2	31.1 ± 6.3	94.5 ± 1.3	15.1 ± 3.3	85.1 ± 8.6	41.0 ± 20.3	84.6 ± 1.7	58.4 ± 3.7
msp	92.9 ± 0.9	47.1 ± 10.3	95.2 ± 1.6	14.0 ± 5.0	88.3 ± 4.1	40.0 ± 14.2	84.6 ± 3.2	61.2 ± 4.8
neco	97.1 ± 1.2	16.4 ± 4.6	99.5 ± 0.2	1.3 ± 1.1	97.5 ± 1.2	13.2 ± 9.6	88.8 ± 2.0	54.6 ± 11.4
residual	97.6 ± 0.8	14.9 ± 5.1	99.6 ± 0.2	1.1 ± 0.9	97.6 ± 1.4	13.0 ± 10.6	89.6 ± 1.6	54.1 ± 8.9
vim	97.6 ± 0.8	14.7 ± 4.4	99.6 ± 0.2	1.1 ± 0.9	97.6 ± 1.4	12.5 ± 10.1	89.9 ± 1.5	53.7 ± 8.6
**Mahalanobis**	**97.6** ± **0.9**	**15.5** ± **5.3**	**99.6** ± **0.2**	**1.0** ± **0.9**	**97.7** ± **1.3**	**12.2** ± **10.0**	**89.8** ± **1.7**	**54.1** ± **9.0**
${\textbf {Mahalanobis}}_{{\textbf {CLS\_AHM}}}$ **(Ours)**	**98.5** ± **0.5**	**7.1** ± **4.1**	**99.7** ± **0.2**	**0.6** ± **0.6**	**97.8** ± **1.2**	**9.9** ± **8.6**	**89.2** ± **1.3**	**52.2** ± **12.6**

**Table 2:** Multi-modal Performance metrics process stage 2 (arithmetic mean and standard deviation) for different methods across two datasets with intra-dataset and cross-dataset experiments configurations per dataset using AUROC (higher is better) and FPR (lower is better)– (cf. Appendix A.1 for hyperparameter tuning details).

Method	Tobacco3482 (ADVE OOD)		Tobacco3482 (Cross-dataset OOD)		FinanceDocs (Resume OOD)		FinanceDocs (Cross-dataset OOD)	
	AUROC (%)	FPR (%)	AUROC (%)	FPR (%)	AUROC (%)	FPR (%)	AUROC (%)	FPR (%)
Mahalanobis_CLS_	97.6 ± 0.9	15.5 ± 5.3	99.6 ± 0.2	1.0 ± 0.9	97.7 ± 1.3	12.2 ± 10.0	89.8 ± 1.7	54.1 ± 9.0
Mahalanobis_Gnome_	97.1 ± 0.9	15.5 ± 5.4	99.2 ± 0.3	3.7 ± 1.6	93.8 ± 3.5	31.4 ± 16.5	82.2 ± 2.4	64.6 ± 11.4
${\textbf {Mahalanobis}}_{{\textbf {AvgAvg}}}$	**94.2** ± **0.8**	**37.5** ± **5.4**	**99.7** ± **0.1**	**0.04** ± **0.05**	**99.6** ± **0.3**	**0.6** ± **0.5**	**94.9** ± **1.5**	**35.3** ± **19.6**

**Table 3:** Multi-modal Performance metrics process stage 1 (arithmetic mean and standard deviation) for different methods across two datasets with intra-dataset and cross-dataset experiments configurations per dataset using AUROC (higher is better) and FPR (lower is better) – (cf. Appendix A.1 for hyperparameter tuning details).

Method	Tobacco3482 (ADVE OOD)		Tobacco3482 (Cross-dataset OOD)		FinanceDocs (Resume OOD)		FinanceDocs (Cross-dataset OOD)	
	AUROC (%)	FPR (%)	AUROC (%)	FPR (%)	AUROC (%)	FPR (%)	AUROC (%)	FPR (%)
knn_CLS_	95.8 ± 1.1	26.9 ± 7.4	99.1 ± 0.4	3.0 ± 1.8	96.5 ± 2.3	17.2 ± 12.7	89.1 ± 1.7	58.9 ± 6.7
${\textbf {knn}}_{{\textbf {CLS\_AHM}}}$ **(Ours)**	**96.9** ± **0.9**	**18.2** ± **3.9**	**99.1** ± **0.3**	**2.4** ± **1.3**	**97.5** ± **1.4**	**11.4** ± **8.8**	**88.5** ± **1.1**	**56.2** ± **9.6**
Mahalanobis_CLS_	97.6 ± 0.9	15.5 ± 5.3	99.6 ± 0.2	1.0 ± 0.9	97.7 ± 1.3	12.2 ± 10.0	89.8 ± 1.7	54.1 ± 9.0
${\textbf {Mahalanobis}}_{{\textbf {CLS\_AHM}}}$ **(Ours)**	**98.5** ± **0.5**	**7.1** ± **4.1**	**99.7** ± **0.2**	**0.6** ± **0.6**	**97.8** ± **1.2**	**9.9** ± **8.6**	**89.2** ± **1.3**	**52.2** ± **12.6**
Mahalanobis_AvgAvg_	94.2 ± 0.8	37.5 ± 5.4	99.7 ± 0.1	0.04 ± 0.05	99.6 ± 0.3	0.6 ± 0.5	94.9 ± 1.5	35.3 ± 19.6
${\textbf {Mahalanobis}}_{{\textbf {AvgAvg\_AHM}}}$ **(Ours)**	**95.6** ± **0.7**	**26.7** ± **0.7**	**99.8** ± **0.1**	**0.01** ± **0.09**	**99.6** ± **0.3**	**0.4** ± **0.3**	**95.1** ± **1.2**	**30.2** ± **1.2**

**Table 4:** Uni-modal Performance metrics process stage 2 for different methods across three datasets using AUROC (higher is better) and FPR (lower is better).

Method	IMDB		News		SST2	
	AUROC (%)	FPR (%)	AUROC (%)	FPR (%)	AUROC (%)	FPR (%)
Mahalanobis_CLS_	99.60 ± 0.05	1.37 ± 0.42	96.14 ± 0.38	19.69 ± 2.36	63.28 ± 1.20	87.14 ± 0.30
${\textbf {Mahalanobis}}_{{\textbf {CLS\_AHM}}}$ **(Ours)**	**99.74** ± **0.06**	**0.52** ± **0.36**	**96.54** ± **0.37**	**17.11** ± **2.45**	**67.51** ± **1.18**	**86.29** ± **0.40**
Mahalanobis_AvgAvg_	99.58 ± 0.02	0.34 ± 0.07	89.56 ± 0.33	57.17 ± 0.36	91.48 ± 0.11	36.40 ± 0.48
${\textbf {Mahalanobis}}_{{\textbf {AvgAvg\_RAND\_AHM}}}$ **(Ours)**	**99.74** ± **0.05**	**0.11** ± **0.09**	**91.58** ± **0.91**	**53.18** ± **2.14**	**93.94** ± **1.38**	**26.16** ± **6.53**
${\textbf {Mahalanobis}}_{{\textbf {AvgAvg\_AHM}}}$ **(Ours)**	**99.78** ± **0.03**	**0.01** ± **0.01**	**92.06** ± **0.80**	**51.24** ± **3.32**	**94.68** ± **0.74**	**23.74** ± **3.89**

**Table 5:** Uni-modal Performance metrics process stage 1 for different methods across three datasets using AUROC (higher is better) and FPR (lower is better), all presented in percentages.

Method	IMDB		News		SST2	
	AUROC (%)	FPR (%)	AUROC (%)	FPR (%)	AUROC (%)	FPR (%)
Mahalanobis_AvgAvg_	99.58 ± 0.02	0.34 ± 0.07	89.56 ± 0.33	57.17 ± 0.36	91.48 ± 0.11	36.40 ± 0.48
${\textbf {Mahalanobis}}_{{\textbf {AvgAvg\_AHM}}}$ **(Ours)**	**99.78** ± **0.03**	**0.01** ± **0.01**	**92.06** ± **0.80**	**51.24** ± **3.32**	**94.68** ± **0.74**	**23.74** ± **3.89**
Mahalanobis_AvgAvg_MC_Drop_	99.79 ± 0.01	0.01 ± 0.01	94.15 ± 0.30	42.51 ± 1.97	95.81 ± 0.13	16.74 ± 0.69
${\textbf {Mahalanobis}}_{{\textbf {AvgAvg\_MC\_Drop\_AHM}}}$ **(Ours)**	**99.87** ± **0.01**	**0.00** ± **0.00**	**95.45** ± **0.50**	**30.65** ± **5.37**	**98.07** ± **0.28**	**8.43** ± **0.97**

### Experimental setup

We employ two widely recognized OOD metrics to assess the performance of our proposed AHM method in comparison to other OOD benchmarks^58^: AUROC, which measures the area under the ROC curve (higher values indicate better performance), and FPR, the false positive rate at a 95% true positive rate. A higher AUROC signifies better discrimination between ID and OOD, while a lower FPR indicates greater robustness in rejecting OOD data.

We set up our experiments to investigate the effectiveness of our proposed AHM method and how it affects the three different stages in the OOD process as shown in Fig. 2 . For our multi-modal experiments, we utilize LayoutLMv3^45^, a transformer-based multi-modal model with 125.92 million parameters. We conduct both cross-dataset and intra-dataset OOD experiments to comprehensively evaluate cases where OOD classes range from very different to more similar to the ID data. In cross-dataset OOD, the model is trained on the classes of one dataset and evaluated on the entirety of the other dataset as OOD. In intra-dataset OOD, one of the 10 classes is designated as OOD, and the model is trained on the remaining 9 classes, with the ID data split into training and evaluation sets. We select Advertisement (ADVE) and Resumes as the OOD classes for Tobacco3482 and FinanceDocs, respectively. For our uni-modal experiments we utilized RoBERTa^44^, a trasnformer-based model of 125 million parameters. For our OOD experiments we consider one of the three datasets as the in-domain data and the remaining two as the ood data. Hence we consider three different settings each time choosing one of the SST-2, IMDB and 20-Newsgroups as the in-distribution dataset and the remaining two as the OOD datasets.

The models for both uni-modal and multi-modal experiments are trained over 5 random runs, with checkpoints saved at high ID classification metrics. Checkpoints with low silhouette scores $s(i) = \frac{b(i) - a(i)}{\max (a(i), b(i))}$ are filtered out to optimize intra-class similarity and inter-class separation where $a(i)$ is the average distance between $i$ and all other points in the same cluster (intra-cluster distance), and $b(i)$ is the average distance between $i$ and all points in the nearest neighboring cluster (inter-cluster distance). Our experiments were conducted using a single NVIDIA A100 GPU (80GB) for 100 GPU compute hours. We trained the models for a maximum of 15 epochs with the hyperparameters outlined in Table S1.

We start our experiments on stage three focusing on the OOD decision mechanisms that discriminates between in-distribution and out-of-distribution data. After choosing a well-performing OOD method, we move on to stage two where we explore different methods for aggregating embeddings generated by the model. After deciding on a well performing aggregation method we move to stage one where our proposed AHM method is applied. Implementing Algorithm 1 with the hyperparameters in Table S2, we identify F attention head masks. These are subsequently used to perform ensemble inference to create embeddings for the OOD task. Finally we set up experiments to explore the performance of our approach (AHM) against other ensembling methodologies, such as MC dropout.^2^

## Overall results

### Stage three and two multi-modal results

For the stage three experiments we evaluated the performance of various OOD detection methods as shown in Table 1. For all the experiments on Table 1 the CLS embedding was used as the stage two aggregation method. Our results show that Mahalanobis^28^ was generally the most performing OOD method, and therefore was chosen for subsequent experiments as the stage three decision mechanism of the OOD process.

**Fig. 4:**
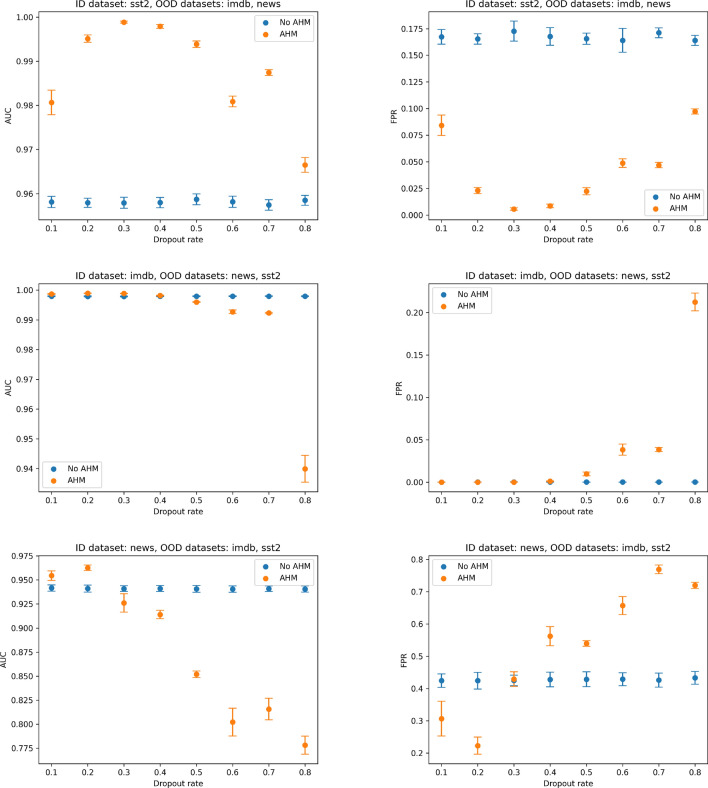
Results of ablation on MC dropout rates. Comparison of AUROC (left column) and FPR (right column) across OOD stages for different uni-modal dataset configurations. For all experiments we use AvgAvg for stage 2 and mahalanabis for stage 3 (Mahalanobis_AvgAvg_). Blue dots represent results obtained with vanilla multi-head-attention (No AHM) where as orange dots represent our proposed AHM.

For Stage two experiments we evaluate different methods for aggregating embeddings such as AvgAvg^3^ and Gnome^17^ against the standard CLS embedding method Mahalanobis_CLS_. As it can been seen on Table 2 the Mahalanobis_AvgAvg_ generally achieved the best AUC and FPR scores demonstrateing the effectiveness of the AvgAvg in achieving more hollistic embedding representations for the OOD task.

### AHM multi-modal results

In Table 3 we compare different methods (KNN+^29^ and Mahalanobis^28^) with different aggregation methods (CLS/AvgAvg) and explore their performance when our proposed AHM method is applied. From the empirical results across the different dataset settings it can be seen that AHM improves both AUC and FPR scores up to 1.4% and 10 % respectively demonstrating the effectiveness of our proposed solution irrespective of the chosen aggregation method at stage two of the OOD process. This is evident through the example shown in Fig. 2 where AHM attenuates spurious dominant signals within the textual representation and thus facilitates accurate identification of the OOD data instance.

### AHM uni-modal results

The results for our uni-modal experiments across three different datasets can be seen in Table 4. As with our multi-modal experiments we assess the effectiveness of our approach under different stage two embedding aggregation strategies (CLS/AvgAvg). From the results obtained it can be seen when AHM is applied that AUC score improves up to 4.2% while FPR score improves up to 12.6%. Furthermore, we evaluate the performance of our method when random attention head masks Mahalanobis_AvgAvg_RAND_AHM_ are chosen against the AHM decided by Algorithm 1 (Mahalanobis_AvgAvg_AHM_). While both the random Mahalanobis_AvgAvg_RAND_AHM_ and the selective Mahalanobis_AvgAvg_AHM_ variants improve over the baseline Mahalanobis_AvgAvg_, Mahalanobis_AvgAvg_AHM_ shows superior performance over the random variant Mahalanobis_AvgAvg_RAND_AHM_ highlighting the effectiveness of Algorithm 1 in identifying optimal AHM for the OOD task.

### AHM vs dropout uni-modal results

In Table 5 we compare the effectiveness of our proposed method against MC dropout by testing Mahalanobis_AvgAvg_MC_Drop_, a method that—similarly to our proposed AHM—can also be applied at stage 1 of the OOD process in Fig. 2. We observe that MC dropout also improves the AUC and FPR scores over the baseline Mahalanobis_AvgAvg_AHM_ but when both MC dropout and our proposed AHM are applied together as Mahalanobis_AvgAvg_MC_Drop_AHM_, we observe the best achievement in both AUC and FPR, indicating that our AHM method can act as a complementary approach to MC Dropout.

### Ablation on MC dropout rates results

Since both MC dropout and AHM effectively shut down neurons at inference time, we conduct further ablation experiments to investigate whether randomly shutting down more and more neurons using MC Dropout is effective on its own, or whether selectively shutting down neurons with AHM can further improve OOD performance. The results of our ablation study can be seen in Fig. 4, showing performance using MC Dropout with AHM (orange) and without AHM (blue). We plot the AUC and FPR scores obtained for the three uni-modal dataset setting under different dropout rates applied at inference time from 0.1 to 0.8.

From the results obtained we can see that just increasing the number of randomly shut down neurons (blue dots) does not have a significant change in the scores obtained. A significant boost in performance for AUC and FPR can be seen when AHM is applied (orange) more specifically between 0.2 and 0.3 dropout rate. For greater dropout rates of 0.3-0.8, the performance with AHM applied starts to deteriorate. Nonetheless, for all dataset settings it can be seen that the optimal performance is achieved when the selective AHM is applied in conjunction with low dropout rates, highlighting the effectiveness of our proposed solution when applied in conjunction with dropout. We further explore the effect of varying the percentage of masking $p$ in Algorithm 1 on FPR and AUROC scores as seen in Figure S1. We observe the optimal value of $p$ to lie within the range of 0.1-0.3.

### Practical computational efficiency considerations

As described in the Computational Efficiency and Scalability section the primary computational cost requirements for our proposed AHM are caused from the Offline Optimization Complexity of Algorithm 1. However, as shown in Table 4, even in the absence of AHM configurations selected via Algorithm 1, randomly sampled AHM ensembles (denoted as Mahalanobis_AvgAvg_RAND_AHM_) consistently outperform the standard Mahalanobis_AvgAvg_ baseline, achieving lower false positive rates across various OOD scenarios. This demonstrates that, even under constrained computational budgets where Algorithm 1 is not feasible, a randomly initialized AHM ensemble with small *K* (e.g., $K=5$) remains effective in enhancing OOD detection performance while maintaining near-real-time inference speeds. This provides a practical trade-off between computational cost and detection accuracy, allowing practitioners to choose an appropriate operating point based on their specific requirements.

## Conclusion

In this work, we introduced Attention Head Masking (AHM), a novel method designed to enhance out-of-distribution (OOD) detection by selectively masking attention heads during inference. Our approach effectively improves the quality of generated embeddings, leading to better separation between in-distribution and OOD data. Through extensive empirical evaluation across both uni-modal and multi-modal settings, we demonstrated that AHM consistently improves AUC and FPR scores, outperforming state-of-the-art methods. Notably, our method remains effective regardless of the chosen embedding aggregation strategy, highlighting its adaptability across different OOD detection pipelines. Furthermore, our ablation studies revealed that selectively shutting down attention heads is more effective than randomly increasing neuron dropout, reinforcing the importance of feature-level control in enhancing OOD detection robustness. Importantly, AHM extends robustly to multimodal document data, reducing the impact of spurious dominant cues within combined textual–visual representations. By introducing FinanceDocs, we aim to foster further research in the document ai domain. Overall, AHM provides a powerful mechanism for improving OOD detection in Transformer-based models, paving the way for more reliable and trustworthy machine learning systems.

## Limitations

While AHM techniques significantly reduced FPR in most cases, the technique limited to attention-based DNN architectures that employ multi-head self-attention. Furthermore the identification of optimal AHM according to algorithm 1 could be further improved rather than using a random sampling method.

## Future work

Given that attention-based architectures leveraging multi-head self-attention mechanisms are currently the predominant models for feature extraction and contextual representation across multiple domains—including vision and speech—our method is broadly applicable. Although our empirical results have primarily been obtained using unimodal text models and multimodal document models, the applicability of the proposed method to other attention-based architectures remains to be thoroughly explored. Future work will investigate the extension of AHM to domains such as speech and additional multimodal settings. Furthermore, to enhance the efficiency and effectiveness of Algorithm 1, we plan to employ model interpretability techniques to analyze the contribution of individual attention heads across layers with respect to in-domain classification performance. By systematically collecting and analyzing activation statistics, we aim to develop a more informed and selective masking strategy that identifies the most salient attention heads. This targeted approach is expected to improve mask selection beyond random sampling, reduce computational overhead, and further enhance OOD detection accuracy.

## Supplementary Information

Supplementary Information.

## Data Availability

The dataset used in this work has been made available at: https://github.com/constantinouchristos/OOD-AHM/tree/main/data For further queries on accessing the data please contact the corresponding author Christos Constantinou.
