# Supplementary material for: Out of distribution detection with attention head masking for multimodal document classification
Source: Sci Rep. 2026 Jan 3;16:2449. doi: 10.1038/s41598-025-32328-9 (PMC12820214; doi:10.1038/s41598-025-32328-9)
Supplement: Supplementary file 1 — Supplementary Information. [file 41598_2025_32328_MOESM1_ESM.pdf]

A

This section provides supplementary material in the form of dataset examples, implementation details, etc. to bolster the reader's understanding of the concepts presented in this work.

A.1 Hyperparameter Tuning

Table S1 summarizes the hyperparameters for model training. The model was trained using a carefully selected set of hyperparameters to optimize its performance. The training batch size per device was set to 32, while the evaluation batch size was configured at 8, ensuring efficient computation throughout the process. To stabilize updates, gradient accumulation was performed over 8 steps. The learning rate was set at 5×10^{-5}, with no weight decay applied, to prevent the risk of overfitting.

The Adam optimizer was configured with parameters $\beta_1 = 0.9$, $\beta_2 = 0.999$, and an epsilon value of 1×10^{-8} to ensure effective convergence. To maintain stability during training, the maximum gradient norm was capped at 1.0. The model underwent training for 15 epochs, with evaluations delayed by 5 steps to monitor progress at appropriate intervals, allowing for a well-tuned and stable learning process.

The hyperparameters chosen for the proposed AHM method are presented in Table S2. Following the procedure outlined in Algorithm 1, an exploration budget of 25 was allocated for potential AHM configurations. To assess the effectiveness of different configurations, masking percentages of 0.1 and 0.2 were applied during the process. The values of 0.1 and 0.2 were chosen following the AHM percentage value ablation experiments performed on the uni-modal data as shown in Appendix A.4

To ensure robust performance, similarity scores between ID validation data and ID training data were computed. These scores were determined by averaging the similarity of the top 10 nearest neighbors for each validation data point. Using these similarity scores, the top five AHM heads were selected to generate the final representation embeddings, which were then combined through an ensemble approach to enhance the overall model performance.

Table S1: Hyperparameters for model training.

**Hyperparameter**	**Value**
per_device_train_batch_size	32
per_device_eval_batch_size	8
gradient_accumulation_steps	8
eval_delay	5
learning_rate	5e-05
weight_decay	0.0
adam_beta1	0.9
adam_beta2	0.999
adam_epsilon	1e-08
max_grad_norm	1.0
num_train_epochs	15

Table S2: Hyperparameters for AHM.

**Hyperparameter**	**Value**
Exploration budget (T)	25
Percentage masking (p)	[0.1, 0.2]
Neighbors (K)	10
Top AHM matrices select (F)	5

A.2 Annotator Training and Validation

To maintain high-quality annotation in line with ethical standards, we enlisted three postgraduate students fluent in English. They received instruction and participated in sessions with finance

professionals to address any task-related questions. The annotation process spanned about four months, involving 90 training sessions, with breaks scheduled every 45 minutes. The students were compensated through gift vouchers and honorariums per minimum wage requirements (see `https://www.minimum-wage.org/international/united-states`).

A.3 Dataset description of FinanceDocs

The FinanceDocs dataset comprises a diverse collection of financial and legal documents sourced from various reliable platforms, offering a comprehensive view of corporate disclosures, shareholder communications, and regulatory filings. Each document type serves a distinct purpose, providing insights into different aspects of corporate governance, financial performance, and regulatory compliance, as detailed below:

- **SEC form documents:** These documents were collected from the Securities Exchange Commission (SEC) website. These forms are statements of changes in beneficial ownership.
- **Shareholder letter documents**: These documents were collected from annual reports. A shareholder letter in an annual report provides a summary of the company's financial performance, highlighting key achievements, strategic initiatives, and market conditions over the past year. It offers leadership's perspective on successes and challenges while outlining future goals and potential risks. The letter also emphasizes the company's commitment to corporate governance, social responsibility, and long-term growth.
- **SEC letter documents:** These documents were collected from the SEC website. These are letters from companies to the SEC about various company disclosures.
- **SEC-13 form documents:** These documents were collected from the SEC website. These forms disclose significant information about an entity's ownership or control over securities, typically required for investors with large holdings.
- **10k form documents:** These documents were collected from annual reports. These represent the 10k forms of an annual report
- **Financial info documents:** These documents were collected from annualreports [51]. They consist of various financial information, including the income statement, balance sheet, and cash flow statement, which detail the company's revenue, expenses, assets, liabilities, and cash movements. It also includes financial ratios and metrics to assess profitability, liquidity, and leverage.
- **Articles of scientific paper documents:** These documents were collected from ACL Anthology `https://aclanthology.org/`. It is a comprehensive digital archive of research papers in computational linguistics and natural language processing, published by the Association for Computational Linguistics.
- **Articles of resume documents:** These documents were collected from Kaggle. They represent resumes from different occupations.
- **Articles of Association documents:** These documents were collected from Companies House Services UK. They represent documents relating to articles of association of a company. These involve information such as directors powers and responsibilities, interpretation and limitation of liability as well as distribution of shares.
- **Director documents:** These documents were collected from annual reports and Companies House Services UK `https://www.gov.uk/government/organisations/companies-house`. It involves information about the directors of a company.

A.4 Exploring different percentage mask values for AHM

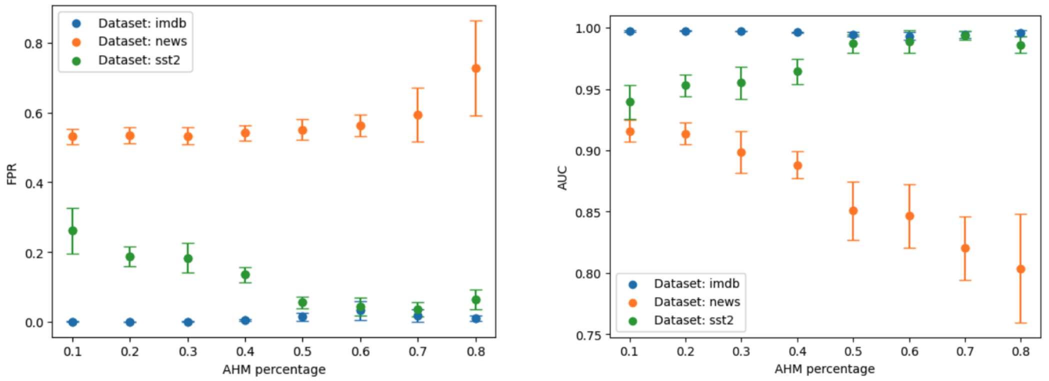

Figure S1: Comparison of AUROC and FPR for different AHM percentage masking values for different dataset configurations.

A.5 Dataset examples of FinanceDocs

Presented below are examples from each document category included in FinanceDocs, providing the reader with a comprehensive visual overview of the dataset.

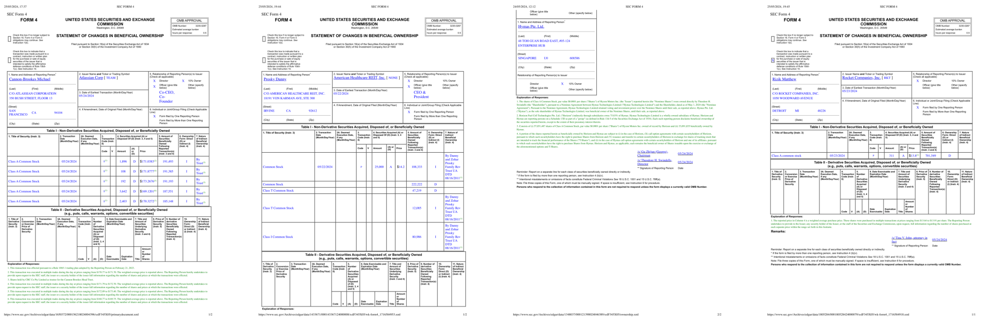

Figure S2: Examples of SEC form documents.

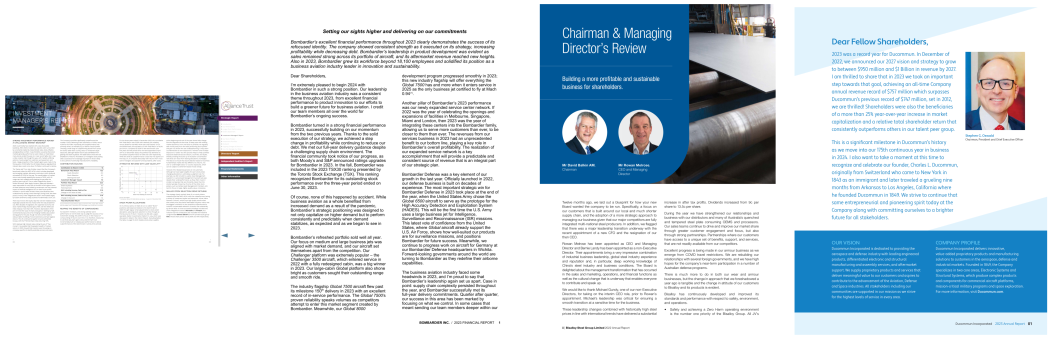

Figure S3: Examples of shareholder letter documents.

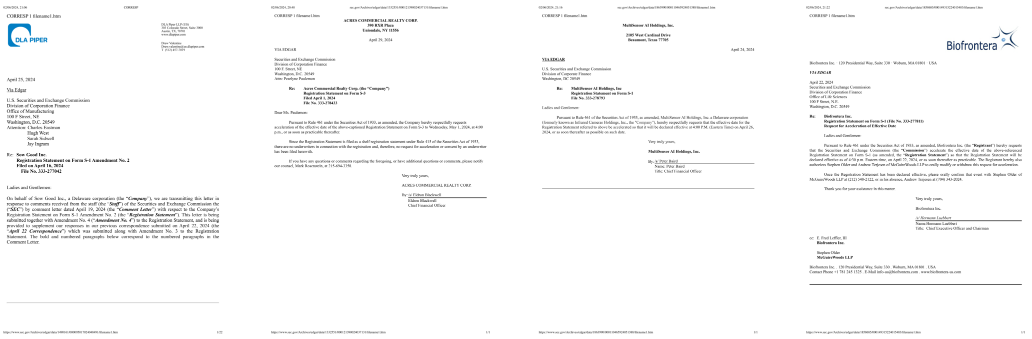

Figure S4: Examples of SEC letter documents.

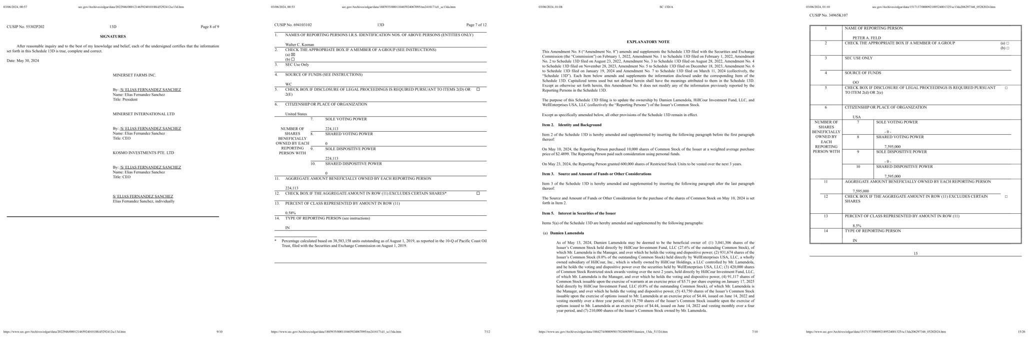

Figure S5: Examples of SEC-13 form documents.

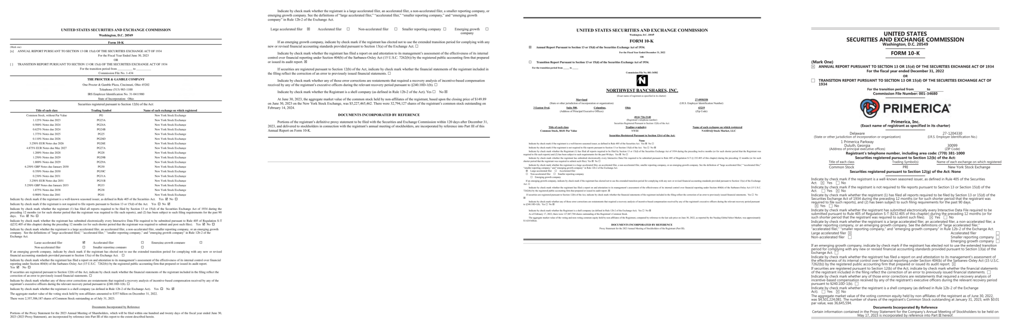

Figure S6: Examples of 10k form documents.

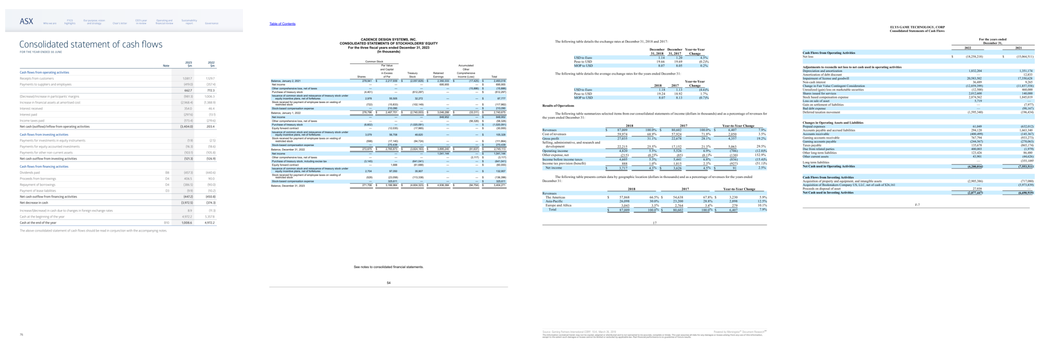

Figure S7: Examples of financial info documents.

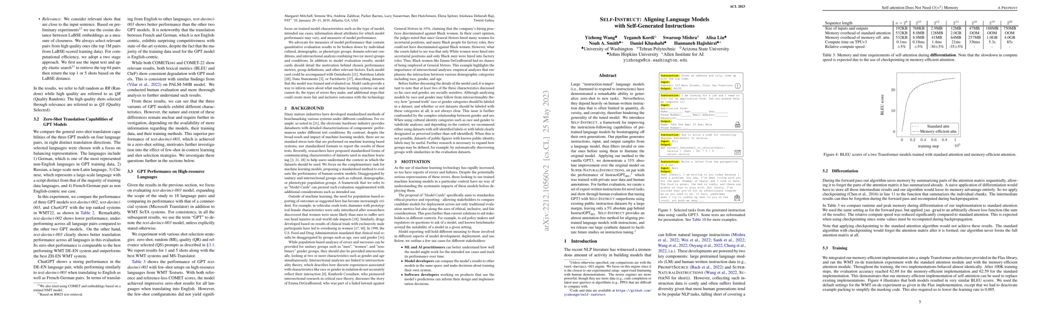

Figure S8: Examples of scientific paper documents.

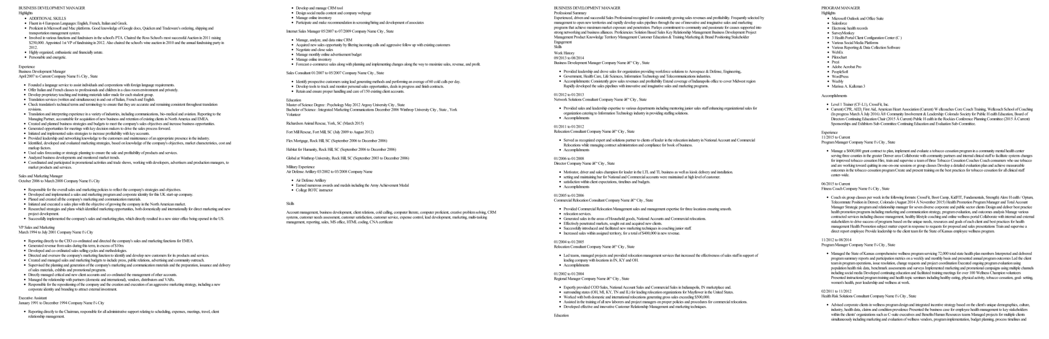

Figure S9: Examples of resume documents.

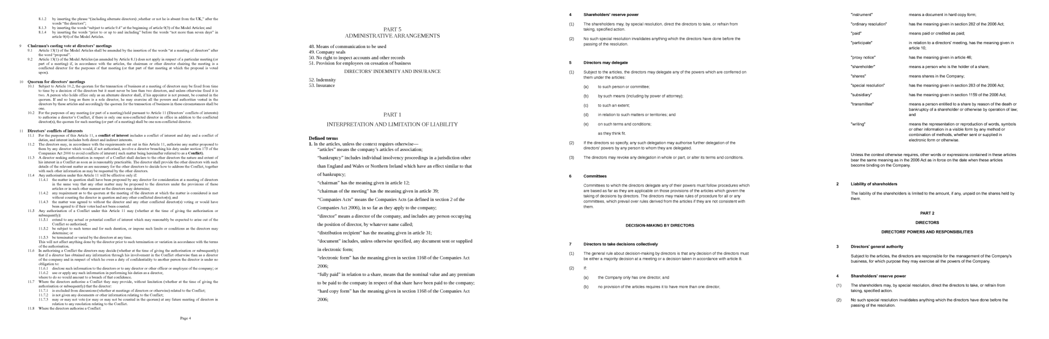

Figure S10: Examples of Articles of Association documents.

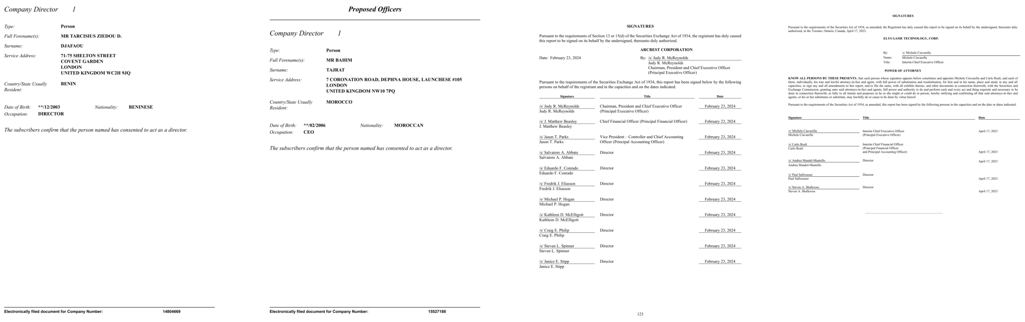

Figure S11: Examples of list of director documents.
